# Psychosocial work environment and antidepressant medication: a prospective cohort study

**DOI:** 10.1186/1471-2458-9-262

**Published:** 2009-07-27

**Authors:** Jens Peter E Bonde, Torsten Munch-Hansen, Joanna Wieclaw, Niels Westergaard-Nielsen, Esben Agerbo

**Affiliations:** 1 Occupational and Environmental Medicine, Copenhagen University Hospital Bisbebjerg, Copenhagen, Denmark; 2 Department of Occupational Medicine, Aarhus University Hospital, Aarhus, Denmark; 3 Center for Corporate Performance, Department of Economics, School of Business, University of Aarhus, Denmark; 4 National Centre for Register-based Research, University of Aarhus, Aarhus C, Denmark

## Abstract

**Background:**

Adverse psychosocial work environments may lead to impaired mental health, but it is still a matter of conjecture if demonstrated associations are causal or biased. We aimed at verifying whether poor psychosocial working climate is related to increase of redeemed subscription of antidepressant medication.

**Methods:**

Information on all antidepressant drugs (AD) purchased at pharmacies from 1995 through 2006 was obtained for a cohort of 21,129 Danish public service workers that participated in work climate surveys carried out during the period 2002–2005. Individual self-reports of psychosocial factors at work including satisfaction with the work climate and dimensions of the job strain model were obtained by self-administered questionnaires (response rate 77,2%). Each employee was assigned the average score value for all employees at his/her managerial work unit [1094 units with an average of 18 employees (range 3–120)]. The risk of first-time AD prescription during follow-up was examined according to level of satisfaction and psychosocial strain by Cox regression with adjustment for gender, age, marital status, occupational status and calendar year of the survey.

**Results:**

The proportion of employees that received at least one prescription of ADs from 1995 through 2006 was 11.9% and prescriptions rose steadily from 1.50% in 1996 to the highest level 6.47% in 2006. ADs were prescribed more frequent among women, middle aged, employees with low occupational status and those living alone. None of the measured psychosocial work environment factors were consistently related to prescription of antidepressant drugs during the follow-up period.

**Conclusion:**

The study does not indicate that a poor psychosocial work environment among public service employees is related to prescription of antidepressant pharmaceuticals. These findings need cautious interpretation because of lacking individual exposure assessments.

## Background

Considerable changes in the work force in affluent countries past 20–30 years have been suggested to increase psychiatric morbidity, in particular depression, because of greater job demands, job insecurity and other job related psychosocial stressors[1]. Some studies have shown that the occurrence of mental disease varies across occupations and the risk of hospital admission for affective and stress-related disorders is above average in the Danish human service professions including health providers, social workers and teachers [2,3]. The job strain model developed by Karasek and Theorell suggests that interaction between perception of high job demands and low job control in terms of skill discretion and decision latitude predicts disease outcomes [4]. Other models with focus on imbalance between work-related efforts and rewards [5] or on organisational and procedural factors [6] have so far received less attention in observational studies addressing risk of mental disorders. A recent meta-analytic review of the scientific evidence provides robust evidence that the individual perception of high demands and efforts and of low decision latitude and rewards are prospective risk factors for common mental disorders [7]. Another systematic review specifically addressing major depression found moderate evidence that individual perception of high psychological demands and low social support at the job increases the risk of depression [8]. It is nevertheless still a matter of conjecture if associations demonstrated in these studies are causal or due to selection bias or confounding [9]. Almost all earlier studies are based upon self-reported data on perceived stressors and health outcome. These are not independent and introduce the risk of circular reasoning and bias because of common method variance [10-12]. This is obvious in cross-sectional studies [1], while it is less acknowledged that prospective follow-up studies do not rule out that associations between perceived stressors and later reporting of depressive symptoms may reflect the way that the individual is perceiving and interpreting the psychosocial working environment[13,14]. For instance, subclinical depression at baseline of follow-up may influence the individuals reporting of psychosocial factors at the workplace. Findings in a recent large Finnish study of workplace social capital emphasize the need to obtain independent exposure assessment. This paper demonstrated moderately elevated risk of depression and prescription of antidepressant medication in relation to workplace social capital at the individual level but not at the aggregated work unit level [15].

The majority of earlier prospective studies addressing job related poor mental health relies on self-reported symptoms and few studies are based upon clinically diagnosed mental disorder [16]. Data on prescription of pharmaceuticals offer an opportunity to obtain independent data on mental health problems that have prompted the individual to seek medical help and that according to a physician require pharmacological treatment. So far only few studies have examined drug prescription in relation to job stress [15,17].

The present study addresses the common method variance problem of many earlier studies on risk of psychiatric disease according to psychosocial workplace factors by independent ascertainment of exposure and outcome. Exposure assessment was based upon the averaged perceived job strain at the work unit level rather than at the individual level while prescription of antidepressant medication was taken as an indicator of poor mental health. The objective was to examine if psychosocial factors at work is related to subscription of antidepressant medications among Danish public service employees.

## Methods

### Study population and data collection

For purposes of a study of work climate, mental and somatic health and sickness absence, we established a Danish cohort of 13,428 employees at Aarhus county and 4,830 employees at Aarhus municipality in 2006–7 as described in details in an earlier paper [18]. In brief, the total of 18,258 employees were addressed in 1,094 workplace questionnaire surveys that were carried out as part of human resource management activities from 1.1.2002 through 31.12.2005. Questionnaires were handed out and subsequently collected at the work place. Absent employees were reached by mail. The surveys were voluntary and anonymous. The response rate was 78.8% in the county and 73.5% in the municipality. Data on departments, job titles, date of work unit entrance and exit (if any) during the follow-up period was obtained from the county and municipality administrations. Demographic data including gender, birth date, area of residence, marital status and number of children less than 15 years were obtained by linking Danish population-based registers using the unique personal identification number, assigned to all persons living in Denmark and used across all registration systems [19]. Individual data on redeemed antidepressants prescriptions from January 1 1995 until December 31 2006 was obtained from the Danish Medicinal Product Registry, which covers all pharmacies in Denmark. Antidepressants are only available by prescription in Denmark. The Danish Data Protection Agency and the Medicinal Product Registry approved the study. Characteristics of the study population are given in Table 1.

**Table 1:** Characteristics of study populations.

Characteristic	County 697 work units 13,335 employees	Municipality 412 work units 4,815 employees
Age at entry, years, mean (min-max)	43.3 (16–71)	37.3 (17–67)
Gender		
Men	21%	23%
Women	79%	77%
Sectors, number of work-units/employees		
Health care	463/9,061	0/0
Social services	115/1,807	362/4,225
Education	74/1,897	50/590
Administration	45/570	0/0
Marital status		
Single	41%	51%
Children at residence	35%	32%

### Psychosocial factors at work

The county surveys were based upon a 40-item questionnaire that was developed as a tool for monitoring and improving psychosocial work conditions [18]. Items were structured as statements with five response categories (yes, very much; yes, somewhat; no, a little; no, not at all; don't know/not relevant) on how well the statement corresponded with their perception of the psychosocial work conditions. The 33 items that were left after omitting seven items on local staff policies clustered around 6 different aspects of psychosocial work conditions: management, work load, skill discretion, decision authority, professionalism and cooperation. The items of these six dimensions are listed in Appendix. The scales were tested for internal consistency (Cronbach's alpha values spanning 0.67 and 0.91), content validity, and discriminant validity [20]. The county questionnaire included also a question on the overall perception of the work climate: "How satisfied are you, all in all, with the psychosocial work conditions at your workplace?" The answers to this question were rated on a scale from 0 (unacceptable) to 10 (exceptional).

The municipalities applied the short form of the Copenhagen Psychosocial Questionnaire (COPQES) that was developed by the Danish National Institute of Occupational Health [21]. This questionnaire includes six items on work demands, 10 items on decision authority and skill discretion combined into one scale measuring decision latitude and 10 items on management and social support. All questions have five response categories (always; often; sometimes; seldom; never or, when appropriate: to a very large extent; to a large extent; partly; to a low extent; to a very low extent). Job strain was defined as the combination of high job demands (upper 25 percentile) and low decision latitude (lower 25 percentile) and isostrain as high job strain in combination with low social support (lower 25 percentile).

The scale values for each respondent were computed by averaging the individual response values (0–3, 4) across all items of a psychosocial dimension and these averages were changed to a 1–100 scale. Subsequently, the arithmetic mean values were computed across respondents in a managerial work unit, and for each dimension (scale), this value was assigned to all members of a work unit, independently of their individual response. This also included the non-respondents of a particular work unit.

COPQES has been evaluated in an earlier publication[21]. The comparability of the COPQES and the county questionnaire with respect to the three job strain dimensions were evaluated by computing correlation coefficients of the corresponding scale-values in 569 respondents that concomitantly answered both questionnaires. All corresponding scale values were significantly correlated. The Spearman coefficients were 0.36 (demands), 0.70 (job control) and 0.82 (social support), respectively.

### Outcome

Antidepressant prescriptions that were redeemed by the study cohort at pharmacies were taken as a proxy measure for affective and stress-related disorders. The Medicinal Product Registry classifies prescribed Pharmaceuticals according to the Anatomical Therapeutic Chemical classification system (ATC) at the level of the generic pharmaceutical. We used prescription of one or more of the following drugs to define the endpoints for the present study: tricyclic antidepressants (TCA, ATC code N06AA), selective serotonin reuptake inhibitors (SSRI, ATC code N06AB), noradrenaline reuptake inhibitors (NARI, ATC code N06AX) and monoamin oxidase inhibitors (MAO-inibitors, ATC codes N06AF and N06AG). Lithium salts are mostly prescribed for bipolar affective disorders and were not included.

### Statistical analyses

We analysed antidepressant drugs redeemed at pharmacies (yes/no) during follow-up according to psychosocial work climate level at baseline using proportional hazard survival regression. Follow-up was measured in days and started the month during which the work climate survey was undertaken and ended when an antidepressant drug was prescribed, the participant left the department or by December 31 2006, whichever came first. Employees who received a prescription of an antidepressant drug during the 6 months preceding the start of follow-up were excluded. In sensitivity analyses this interval was extended to one year, two years and three years. The dimensions of the psychosocial work environment were categorised by quartiles and entered into the models as dummies of the upper quartile and the two intermediate quartiles using the lowest quartile (best work environment) as reference. Each work climate scale was entered separately. Models were adjusted for a fixed set of potentially confounding factors that were identified prior to data analyses as having known or suspected causal relation to affective disorders. These factors were introduced as dummy variables: gender (male yes/no), age at start of follow-up (41–50, 51–60, >60 with <41 as implicit reference category), marital status (cohabitating, yes/no), children less than 15 years at the residence (yes/no) and socio-economic status (low, medium or high with the latter as reference category). Occupational status as an indicator of socio-economic class was categorized into three levels based upon the occupational code [Danish version of the International Standard Classification of Occupation 1988 (ISCO88) of the International Labor Office (ILO, 1990)] using Erikson and Goldthorpe's class categories [22]. Hazard ratios (HR) and robust 95% confidence limits based on the work units were computed using the SAS 9.1 software (PHREG procedure) [23].

## Results

The 21,129 county and municipality employees purchased a total of 789,843 prescribed pharmaceuticals from January 1 1996 through December 31 2006. Antidepressant drugs with the ATC code N06A comprised 45,141 prescriptions (5.7% of all) and 66% of these were SSRI compounds, 22% NARI compounds and 12% TCA compounds. Less than 0.3% was monoamine oxidase inhibitors (ATC codes N06AG and N06AF). The proportion of employees that received at least one prescription of antidepressant drugs from 1995 through 2006 was 11.9%. The purchase of antidepressant drugs in the entire study population rose steadily from 1.5% in 1996 to the highest level 6.5% in 2006. The average number of redeemed prescriptions per employee with at least one prescription was 17.9 (range from 1 to 201), but only 45% received more than one prescription of antidepressant drugs.

None of the measured dimensions of the work climate in the county population were related to redeemed prescriptions of antidepressant drugs during the follow-up period in either crude models or models adjusted for effects of gender, age, education, civil status and calendar year of the workplace survey (Table 2).

**Table 2:** Adjusted hazard ratio (HR_adj_) for purchase of prescribed antidepressant drugs according to six dimensions of the work climate at the managerial work unit level categorised into quartiles (percentiles 0–25, 26–75 and 76–100) in 13,335 public service workers in Aarhus county 2002–2006.

**Psychosocial work characteristic**	**Range of average work unit score** **(1–100)**	**N cases**	**Person years** **at-risk**	**HR**_adj_	**95% CI**
Overall work climate satisfaction					
Low	29–66	129	7,547	0.92	0.72–1.17
Intermediate	67–78	302	15,576	1.09	0.88–1.33
High (reference)	79–96	143	7,996	1.00	Reference
Appropriate management					
No	17–49	114	7,705	0.80	0.62–1.02
Limited	50–57	310	15,817	1.00	0.82–1.22
Yes (reference)	58–86	150	7,596	1.00	Reference
Appropriate work load					
No	25–64	164	7,604	1.09	0.88–1.36
Somewhat	65–76	252	15,721	0.83	0.68–1.01
Yes (reference)	77–98	158	7,793	1.00	Reference
Appropriate skill discretion					
No	45–72	147	7,825	1.07	0.85–1.36
Somewhat	73–82	294	15,410	1.12	0.91–1.37
Yes (reference)	83–100	133	7,883	1.00	Reference
Appropriate decision authority					
No	41–67	139	7,627	1.10	0.87–1.40
Somewhat	68–77	304	15,688	1.19	0.97–1.47
Yes (reference)	78–98	130	7,765	1.00	Reference
Appropriate professionalism					
No	37–68	129	7,717	0.96	0.76–1.21
Somewhat	69–79	290	15,721	0.98	0.80–1.19
Yes (reference)	80–97	155	7,680	1.00	Reference
Appropriate cooperation					
No	32–61	136	7,275	0.87	0.69–1.11
Somewhat	62–73	284	15,731	0.97	0.79–1.18
Yes (reference)	74–94	154	8,039	1.00	Reference

While job demands, decision latitude, job strain and isostrain was not related to purchase of antidepressant medication the highest level of poor social support at work was associated with an increased risk among employees in the municipality (Table 3). No indication of exposure response was found.

**Table 3:** Adjusted hazard ratio (HR_adj_) for purchase of prescribed antidepressant drugs according to the job strain at the managerial work unit level categorised into quartiles (percentiles 0–25, 26–75 and 76–100) in 4,815 public service workers in Aarhus Municipality 2002–2006.

**Psychosocial work characteristic**	**Range of average work unit score** **(1–100)**	**N cases**	**Person years** **at-risk**	**HR**_adj_	**95% CI**
High job demands					
Yes	10–50	81	2,909	1.16	0.84–1.59
Somewhat	51–60	175	5,581	1.27	0.96–1.67
No (reference)	61–96	72	2,852	1.00	Reference
Low decision latitude					
Yes	48–64	97	2,766	1.24	0.92–1.67
Somewhat	65–70	152	5,725	0.94	0.71–1.23
No (reference)	71–86	79	2,852	1.00	Reference
Low social support					
Yes	26–58	104	2,795	1.50	1.11–2.03
Somewhat	59–67	153	5.689	1.08	0.81–1.43
No (reference)	68–88	71	2,858	1.00	Reference
High job strain^1^					
Yes	.	36	1,087	1.19	0.84–1.68
No (reference)	.	292	10,256	1.00	Reference
High isostrain^2^					
Yes	.	22	674	1.17	0.76–1.80
No (reference)	.	306	10,669	1.00	Reference

^1 ^high job demands (upper 25 percentile) and low decision latitude (lower 25 percentile)

^2 ^job strain combined with low social support (lower 25 percentile)

None of the associations were modified by gender and therefore results are not presented separately for men and women. All analyses were repeated among the 45% of cases who received more than one prescription of an antidepressant. Findings were similar. Differences between model-based and robust sandwich estimates were marginal affecting the third or fourth decimal of the estimate. All covariates contributed significantly to the regression model. Thus antidepressants were prescribed more frequent among women (HR 1.3, 95% CI 1.01–1.56), middle aged 41–50 years (HR 1.8, 95% CI 1.34–2.42), employees with low occupational status (HR 1.3 95% CI 1.07–1.57), those living alone (HR 1.4, 95% CI 1.2–1.69) and in more recent calendar years.

## Discussion

This prospective study of a large population of Danish public service employees with complete follow-up does not provide evidence that the psychosocial work environment is a major contributor to psychiatric morbidity. With the exception of social support none of the measures of psychosocial workplace factors were related to redeemed prescriptions of antidepressant pharmaceuticals during up to four years of follow-up. Poor social support at the workplace was related to increased rate of purchase of antidepressants but this association was only observed in the municipality and was not consistent with findings in the county. Therefore this finding should not be given much weight.

Selection bias is hardly an issue in this study. A little less than 80% of employees filled in the questionnaire on work-related psychosocial factors but follow-up data on antidepressant prescriptions were collected in the registries for all employees regardless of whether the filled in the questionnaire or not. The lacking exposure information from 1/5 of the population has not introduced biased findings since sensitivity analyses omitting work-units with low response rates produced essentially the same results. Unfortunately we have no information on how often patients get a prescription of antidepressants by their doctor without actually getting the medicine. Similarly, it is not known how often redeemed medicine is actually taken as prescribed by the doctor.

Our measure of job exposure can be considered to be somewhere in-between measures based upon job exposure matrices and measures based upon self-reported exposures. Studies based on job titles have rarely demonstrated positive associations between psychosocial job stressors and ill mental health while several studies using self-reports have reported positive associations to depressive disorders. [9] The lack of associations between aggregated measures of exposure and prescription of antidepressant pharmaceuticals in the present study does unfortunately not help to solve the fundamental problem: are job exposure matrix studies erroneously negative because of dilution of exposure by assigning average exposure levels in entire job categories [24-26] or areself-report studies erroneously positive because of circular reasoning the exposure and outcome not being measured independently. The latter alleges that predisposition to develop a depressive disorder is related to a less favourable perception of the work environment in general [10]. Besides intervention trials it seems prudent to develop new methodologies of feasible independent measures of individual exposure to psychosocial strain. In any case, it must be acknowledged that psychosocial strain at the individual level, such as threats and violence, [27] may be poorly reflected by an aggregated measure of exposure as used in this study.

According to the cognitive activation theory of stress (CATS) the individual experience of the stressor and the experience of the stress response are important components of the stress process that – when sustained over time – may increase the risk of disease [28]. It can be argued that any attempt to measure stressors and stress-outcomes independent of the individual perception will miss the point and throw out the baby with the bath water. It is evident that the diagnosis of psychiatric morbidity to a large extent will rely on subjective reports, but so much the more is it important to obtain independent measures of stressors as long as the objective is to unravel causal relations that may pave the way for preventive interventions. This does not contradict that subjective perceptions are important in the pathogenesis of stress-related diseases exactly as we don't need to know about the individual metabolism of benzene in order to study the risk of leukemia in exposed workers.

Although the component of exposure variation between work units only accounted for 14–35% of the total variation, the exposure contrast across the more than one thousand managerial work units was pretty high (Table 2 and 3). It is acknowledged that misclassification of exposure is an important issue that usually will produce bias toward the null but in this study the average exposure levels in the uppermost and lowest exposure quartiles varied by a factor 2–3 and therefore exposure misclassification seems of minor importance.

Lack of associations could reflect focus on inappropriate dimensions of the psychosocial work environment. For instance we did not explicitly measure effort-reward imbalance, organisational injustice or work related violence that in some studies have been associated with ill mental health. [9] However, there is a lack of international consensus on how to measure the psychosocial work environment and workplace surveys that were used include most psychosocial factors that are thought to be of importance. Moreover, findings in the county and the municipality that applied two different questionnaires were consistent.

We computed the average psychosocial work environment scores for each work unit and assigned these values to all employees working in that unit to obtain more independent measures of exposure. The statistical analysis accounted for the artificially diminished exposure variation, which may cause too narrow confidence limits, by robust sandwich estimating methods as described by Lin and Wei.[23] Although the examined associations probably are attenuated as a result of aggregation [29], ecological or aggregated information in individual-based studies can generally be useful in identifying causal relationships [30].

Prescription of SSRI drugs was more than five-doubled in the study population during the decade from 1996 onwards, while prescriptions of anxiolytica as benzodiazepins and hypnotics during the same period were stable or slightly increasing. This parallels prescription of antidepressants in entire Denmark during the same time period [31]. Since the workplace surveys were performed consecutively from 2002 through 2005 it was important to adjust risk estimates for calendar time of the survey. Adjustments for other factors that all were highly significantly related to prescription of drugs only changed the risk estimates marginally. These factors included gender, age, occupational status and marital status and the strong associations between these variables and prescription of antidepressant drugs corroborates an earlier Danish report [32]. However, in light of the lack of associations between work climate and prescription of antidepressant drugs observed in this study positive confounding is less of an issue.

In an earlier study, we observed higher risk for hospital referral for affective or stress-related disorders among human service professions that to a large degree were addressed by the present study [2,3]. These findings are not discrepant with results of the present study because an increased occurrence among members of certain occupations is not always, of course, due to occupational exposures.

In accordance with recent results of a nationwide Danish study [31], we found that 55% of employees that were prescribed an antidepressant medication stopped treatment after the first prescription. SSRIs have no documented effect on less severe cases of depression and 25–50% of moderate and severe cases do not respond to pharmaceutical treatment. This may explain part of the high rate of discontinuance after the first prescription. Furthermore, even in cases that respond to medical treatment it takes some 2–4 weeks before effects are seen. In Denmark 6–12 months of treatment is recommended for first-occurrence of a depressive episode and longer treatments are recommended for recurrent cases. Subgroups of patients that are distinguished by duration of treatment could have different diseases with different causes. However, reanalyses of each of these large subsets did still not reveal any association with the work climate.

The new generation of SSRI and NARI pharmaceuticals are far from exclusively prescribed for affective disorders but also for stress-related and anxiety disorders which, however, is considered less of a problem relative to the objective of this paper since the hypotheses on work related psychiatric morbidity are not exclusively focused on affective disorders [7]. There are, however, other medical indications for prescription of these drugs as well [33] and it must be acknowledged, that prescription of drugs is a crude proxy for disease occurrence and also reflect access to health care, illness behaviours and treatment conventions.

## Conclusion

The study does not indicate that a poor psychosocial work environment among public service employees is related to prescription of antidepressant pharmaceuticals. However, the findings need cautious interpretation because of lacking individual exposure assessments.

## Competing interests

The authors declare that they have no competing interests.

## Authors' contributions

JPB conceived and designed the study, performed statistical analyses and drafted the manuscript. TMH developed the county questionnaire, coordinated data collection and established the cohort, EA contributed significantly to the statistical analysis and drafting of the paper. JW and NWN contributed to design and interpretation of findings. All authors contributed to the revised manuscript, read and approved the final manuscript.

## Appendix. Items clustering about six dimensions of the psychosocial work environment. Aarhus County 2002–07

(1) Management (Cronbachs α = 0.91)

• The management is motivating and inspiring

• The management swithces appropriately between listening and talking

• The management has a good grasp of the workplace

• It pays off to talk to the management about difficulties in my work

• Conflicts are solved appropriately at the workplace

• The manager is capable of decisive action

• The management gives me favorable and critical comments in a way that motivates me to improve my work

• My effort is appreciated

(2) Skill discretion (Cronbachs α = 0.84)

• I am satisfied with the challenges I get through my work

• The work gives me the opportunity to exercise my personal and professional skills

• My job is exciting

• My work is appropriately varied

• My workplace gives me the opportunity for personal and professional development

(3) Cooperation (Cronbachs α = 0.85)

• I feel comfortable with the rhetoric and the social conventions at the workplace

• I find that the workplace is characterized by a cooperative spirit

• We treat each other as equals and with respect

• We support each other in difficult situations at work

• At the workplace we give each other favorable and critical comments in a way that motivates me to improve my work

• In my experience there is equality of opportunity at my workplace (across e.g. age, gender or ethnicity)

• Everybody can say his opinion freely at my workplace

(4) Workload (Cronbachs α = 0.67)

• I can combine the demands at work with a sound private life

• I am satisfied with my daily amount of work

(5) Professionalism (Cronbachs α = 0.86)

• The products of my workplace are of high quality

• There is agreement between our daily work and our values and goals

• In my experience there is agreement about the values and goals of the workplace

• At the workplace we are preoccupied by improving the quality of the work

• Time and energy are deployed for the right purposes

• My workplace enjoys respect among users and collaborators

• If something doesn't work it is addressed appropriately

(6) Decision Authority (Cronbachs α = 0.69)

• I find that the employees are involved appropriately when decisions are made

• I am satisfied with my opportunity to influence the planning of my work

• I have the information I need to perform my work

• I know what is expected from my work

## Pre-publication history

The pre-publication history for this paper can be accessed here:


